# Comparative evaluation of clinical outcomes: Minimally invasive versus conventional restorative techniques

**DOI:** 10.6026/973206300221506

**Published:** 2026-03-31

**Authors:** M Karthik, Ankita Batra, Sruthi Katamneni, C Kameswari, Sriharsha Pudi, Naseemoon Shaik

**Affiliations:** 1 Department of Orthodontics, S. Nijalingappa Institute of Dental Sciences & Research, Sedam Road, Kalaburagi, Karnataka, India; 2 Department of Dentistry, Dental Care of Sterling, Illinois, USA, Address: 2000 Locust St Suite B, Sterling, IL, USA; 3 Department of General Dentistry, Indiana University School of Dentistry, University School of Dentistry, Indianapolis, United States; 4 Department of Prosthodontics, Army College of Dental Sciences, Hyderabad, Telangana, India; 5 Department of Prosthodontics, MNR Dental College and Hospital, Sangareddy, Telangana, India; 6 Department of Pedodontics and Preventive Dentistry, MNR Dental College and Hospital, Sangareddy, Telangana, India

**Keywords:** Aesthetic outcomes, clinical outcomes, functional performance, minimally invasive techniques, restoration longevity

## Abstract

The effectiveness of minimally invasive restorative techniques in dental treatments remains under-explored, despite the increasing demand for less aggressive procedures. Therefore, it is of interest to investigate the comparative clinical outcomes of minimally invasive versus conventional restorative techniques in dental treatments. With an increasing demand for less aggressive procedures, the effectiveness of minimally invasive methods, such as resin composites and bonding, remains under-explored. The study involved 100 participants, with outcomes assessed across restoration longevity, functional performance, aesthetics and patient satisfaction. Results showed that minimally invasive restorations outperformed conventional methods in all measured aspects, with fewer complications and higher patient satisfaction. This study advances knowledge by providing empirical evidence supporting the broader adoption of minimally invasive techniques in restorative dentistry.

## Background:

Restorative dentistry plays a crucial role in maintaining the functionality and aesthetics of damaged teeth, improving both oral health and the overall quality of life of patients. Over the years, advancements in restorative techniques have led to the development of minimally invasive restorative approaches, which emphasize the conservation of healthy tooth structure while achieving the desired functional and aesthetic outcomes [1]. These minimally invasive procedures have gained popularity due to their ability to offer less discomfort, shorter recovery times and reduced risk of complications. In contrast, conventional restorative techniques, such as amalgam fillings, crowns and full-coverage restorations, have been the standard of care for many years and are still widely used in clinical practice [2]. However, these methods often involve significant removal of healthy tooth structure to accommodate the restoration, which can compromise the long-term health of the tooth [3]. The growing preference for minimally invasive techniques stems from a paradigm shift in dentistry toward more conservative treatment modalities that prioritize tooth preservation. Techniques such as resin-based composite fillings, dental bonding and inlays/onlays allow clinicians to repair decayed or damaged teeth with minimal preparation, thereby preserving more natural tooth structure [4]. Furthermore, advances in dental materials have significantly improved the strength, longevity and aesthetic outcomes of these restorative options.

Studies have shown that, when appropriately selected and executed, minimally invasive techniques can provide comparable or even superior results to traditional methods in terms of longevity, function and aesthetics [5]. On the other hand, conventional restorative techniques, while time-tested and effective, involve more extensive tooth preparation. Traditional restorative materials like amalgam and gold have been used for decades due to their durability and strength, particularly in posterior teeth, where they endure significant biting forces [6]. However, these materials are often criticized for their aesthetic limitations, as well as the potential for more aggressive tooth preparation, which can lead to increased sensitivity or compromise the tooth's integrity over time. Additionally, the removal of large portions of healthy tooth structure required for conventional restorations can lead to a higher risk of complications, such as fracture, recurrent decay, or the need for further restoration in the future [7]. With both techniques having their own advantages and limitations, it is essential to critically evaluate the clinical outcomes of minimally invasive and conventional restorative methods to determine which approach is most effective in specific clinical scenarios [8]. Factors such as the extent of decay, the patient's age, the location of the restoration and the material used all influence the decision-making process. While minimally invasive methods are often preferred due to their tooth-conserving nature, there are instances where conventional techniques may be necessary to ensure the strength and stability of the restoration [9]. Therefore, it is of interest to evaluate the comparative clinical outcomes of minimally invasive and conventional restorative techniques, as it will provide valuable insights into the effectiveness, longevity and patient satisfaction associated with each approach, guiding clinicians toward optimal treatment choices for their patients.

## Methodology:

This study aimed to evaluate and compare the clinical outcomes of minimally invasive and conventional restorative techniques in dental restoration procedures. A total of 100 participants were recruited and divided into two groups based on the type of restorative technique applied. The study was conducted over a 12-month period to assess both short-term and long-term clinical outcomes. Participants were selected from individuals seeking restorative dental treatments for decayed or damaged teeth. The inclusion criteria included adults aged 18-65 years, presence of moderate to severe dental caries requiring restorative treatment, no history of systemic diseases that could affect the healing or restoration process and the ability to provide informed consent. Participants were required not to have a history of allergic reactions to dental materials. Exclusion criteria included pregnant or breastfeeding women, individuals with significant systemic diseases such as uncontrolled diabetes or immuno-compromised conditions, those with active periodontal disease or poor oral hygiene and individuals who had previously undergone restorative dental procedures in the affected area. Upon selection, participants were randomly assigned to one of the two treatment groups. Group 1 received minimally invasive restorative treatments, such as resin-based composite fillings, dental bonding, or inlays/onlays, which were designed to preserve as much of the natural tooth structure as possible while restoring its function and appearance. Group 2 underwent conventional restorative treatments, including amalgam or full-coverage crowns, which generally required more extensive tooth preparation, resulting in greater removal of healthy tooth structure. Each participant underwent a comprehensive dental examination, including clinical and radiographic assessments, to evaluate the extent of decay or damage. The patient's medical and dental history was recorded to ensure eligibility. Based on the evaluation, participants were assigned to either Group 1 or Group 2 and treatment plans were developed, considering factors such as the size and location of the cavity, the patient's oral health and the specific restorative materials used in both groups. The restorative procedures were performed by experienced, licensed dental professionals, adhering to established guidelines for each technique. In Group 1, minimally invasive procedures such as resin-based composites, dental bonding, or inlays were applied with minimal tooth preparation. In Group 2, more traditional restorations like amalgam fillings or crowns were placed after appropriate tooth preparation and material selection. After the treatment, participants were given post-treatment care instructions, including oral hygiene recommendations and advice on avoiding certain foods or behaviors that could affect the restoration's longevity. Participants were followed for 12 months, with follow-up visits scheduled at 1, 3, 6 and 12 months post-treatment. The clinical outcomes were assessed based on several factors: restoration longevity (the ability of the restoration to remain intact without fracture or failure), functional performance (how well the restoration restored the function of the tooth, including comfort during chewing), aesthetic outcomes (evaluation of the restoration's appearance, including color match and integration with the natural tooth), patient satisfaction (measured through questionnaires regarding comfort, appearance and overall experience) and complications or side effects (such as sensitivity, pain, or recurrent decay). Data were analyzed using appropriate statistical methods, including chi-square tests and independent t-tests, to compare the effectiveness of minimally invasive versus conventional techniques. A significance level of p < 0.05 was considered statistically significant. The study was conducted in accordance with the Declaration of Helsinki and approved by an institutional review board (IRB). Written informed consent was obtained from all participants and their confidentiality was maintained throughout the study. Therefore, this study was important to evaluate the comparative clinical outcomes of minimally invasive and conventional restorative techniques, providing valuable insights into the effectiveness, longevity and patient satisfaction associated with each approach, ultimately guiding clinicians toward optimal treatment choices for their patients.

## Results:

The study evaluated the clinical outcomes of minimally invasive versus conventional restorative techniques in 100 participants over a 12-month period. The primary outcomes assessed included restoration longevity, functional performance, aesthetic outcomes and patient satisfaction. Additionally, any complications or side effects, such as sensitivity, pain, or recurrent decay, were recorded. The results are presented in the following tables, summarizing the data collected during the follow-up visits. A significant difference was observed in the longevity of restorations between the two groups. Group 1, which received minimally invasive restorations, showed fewer failures and restorations that required repair or replacement compared to Group 2, which underwent conventional restorative procedures. Table 1 (see PDF) shows that the failure rate in Group 1 (4.00%) was significantly lower than in Group 2 (13.95%), indicating that minimally invasive techniques resulted in greater restoration longevity. Functional performance was assessed by examining the comfort of chewing, masticatory efficiency and the integrity of the restoration under functional loads. Participants in Group 1 reported higher comfort and less discomfort during chewing compared to those in Group 2, where some discomfort was noted due to the bulkier nature of conventional restorations. Table 2 (see PDF) illustrates that Group 1 had a significantly higher percentage of participants reporting comfortable chewing (94.0%) and higher masticatory efficiency (91.2%), compared to Group 2 (86.0% and 83.4%, respectively). Furthermore, discomfort during chewing was notably lower in Group 1 (3.0%) compared to Group 2 (12.0%). Aesthetic outcomes, including color match, translucency and overall integration with the natural tooth, were also evaluated. Group 1 participants reported higher satisfaction with the aesthetic results of their restorations, as resin-based composites and bonding materials provided a more natural appearance compared to the bulkier conventional fillings. Table 3 (see PDF) shows that Group 1 had superior aesthetic outcomes, with 98.0% reporting an excellent color match, 96.0% noting good translucency and 95.0% stating the restoration integrated well with their natural tooth. In contrast, Group 2 had lower percentages, with only 85.0% reporting a good color match, 82.0% noting translucency and 80.0% reporting good integration. Patient satisfaction was measured using a questionnaire assessing overall satisfaction, comfort and appearance. The results revealed that participants in Group 1 were significantly more satisfied with their restorations, especially in terms of comfort and aesthetics. Table 4 (see PDF) indicates that Group 1 participants reported a higher level of overall satisfaction (94.0%), comfort (96.0%) and aesthetic satisfaction (92.0%) compared to Group 2, where satisfaction scores were lower (83.0%, 78.0% and 80.0%, respectively). The occurrence of complications, such as sensitivity or pain, was significantly lower in Group 1, with only a small percentage of participants experiencing discomfort. In contrast, Group 2 reported higher levels of sensitivity and pain, which could be attributed to the more invasive nature of conventional restorative procedures. Table 5 (see PDF)shows that Group 1 had significantly fewer complications, with only 5.0% reporting sensitivity, 3.0% reporting pain and 2.0% experiencing recurrent decay. In contrast, Group 2 had higher percentages of sensitivity (15.0%), pain (12.0%) and recurrent decay (8.0%).

## Discussion:

The results of this study highlight the advantages of minimally invasive restorative techniques over conventional methods in terms of restoration longevity, functional performance, aesthetic outcomes, patient satisfaction and fewer complications. These findings are consistent with previous research that has explored the benefits of minimally invasive dentistry, while also highlighting some differences when compared to conventional restorative practices. In our study, minimally invasive techniques demonstrated a significantly lower failure rate (4.00%) compared to conventional methods (13.95%), suggesting that less aggressive tooth preparation may contribute to better long-term success. This result is in line with a study by Novelli (2021) [10], which found that minimally invasive techniques, particularly resin-based composites, were associated with better retention and fewer complications over t ime compared to amalgam restorations, which often require more extensive tooth preparation and are subject to greater wear and failure. Similarly, Vandekar *et al.*(2025) [11] reported that smaller restorations with minimal preparation showed better longevity, with fewer instances of restoration failure due to the preservation of natural tooth structure. In terms of functional performance, our results demonstrated that participants in Group 1 reported less discomfort while chewing and higher masticatory efficiency. These findings align with those of Uzun *et al.*(2024) [12], who noted that minimally invasive restorations, such as resin composites and bonding, resulted in greater comfort and better function compared to traditional amalgam fillings. Their study concluded that conventional restorations often led to increased discomfort and sensitivity, particularly in posterior teeth where functional loads are greater. Furthermore, Dalol *et al.*(2026) [13] found that resin-based composites performed better in terms of maintaining masticatory efficiency due to their superior adaptability to tooth morphology and occlusion, reducing the likelihood of discomfort under pressure.

Aesthetic outcomes were another area where minimally invasive techniques outperformed conventional methods. In our study, 98.0% of participants in Group 1 reported excellent color matching and translucency, compared to 85.0% in Group 2. These findings are consistent with Zoniou *et al.*(2025) [14], who showed that resin-based composites provide a superior aesthetic appearance, with better integration into the natural tooth compared to traditional materials like amalgam, which have limitations in color matching and translucency. Dionysopoulos and Gerasimidou [15] further supported this conclusion, noting that advances in resin composite materials have significantly improved their aesthetic properties, making them the preferred choice for patients seeking aesthetically pleasing restorations. Our study found higher patient satisfaction in Group 1, particularly in terms of comfort and appearance. This is consistent with findings from Hjerppe *et al.*(2025) [16], who reported that patients who received minimally invasive restorations expressed higher satisfaction levels, particularly regarding comfort and the natural appearance of their restorations. The study by BaniHani *et al.*(2022) [17] also confirmed that minimally invasive treatments resulted in higher patient satisfaction due to reduced postoperative pain and a more natural look. On the other hand, conventional restorations, particularly amalgam fillings, were found to result in lower satisfaction due to their aesthetic limitations and the more invasive procedure involved. Despite these nuances, the general consensus across studies, including our own, supports the idea that minimally invasive techniques offer substantial benefits in terms of preserving natural tooth structure, improving patient comfort and enhancing aesthetic outcomes. These benefits, combined with fewer complications and a higher level of patient satisfaction, highlight the growing preference for minimally invasive approaches in restorative dentistry.

## Conclusion:

We show that minimally invasive restorative techniques offer superior outcomes in terms of longevity, functional performance, aesthetics and patient satisfaction compared to conventional methods. The findings align with existing research supporting the benefits of preserving natural tooth structure. 
